# Transdermal versus oral estrogen: clinical outcomes in patients undergoing frozen-thawed single blastocyst transfer cycles without GnRHa suppression, a prospective randomized clinical trial

**DOI:** 10.1007/s10815-018-1380-5

**Published:** 2018-12-05

**Authors:** Semra Kahraman, Caroline Pirkevi Çetinkaya, Yucel Sahin, Gokalp Oner

**Affiliations:** Assisted Reproductive Technologies and Reproductive Genetics Centre, Istanbul Memorial Hospital, Piyale Pasa Bulvari, 34385, Okmeydani, Istanbul, Turkey

**Keywords:** Oral estrogen, Transdermal estrogen, Endometrial thickness, Cycle outcomes

## Abstract

**Purpose:**

To conduct a non-inferiority study to compare the clinical outcomes of transdermal estrogen patch and oral estrogen in patients undergoing frozen-thawed single blastocyst transfer non-donor cycles without GnRHagonist (GnRHa) suppression.

**Methods:**

A total of 317 women with irregular menses or anovulatory cycle undergoing frozen-thawed embryo transfer (FET) non-donor cycles without GnRHa suppression were involved in a prospective randomized clinical trial between May 2017 and October 2017. The trial was conducted in an ART and Reproductive Genetics Centre within a private hospital. The unit is designated as a teaching center by the Turkish Ministry of Health. Oral or transdermal estrogen was administered in patients undergoing frozen-thawed single blastocyst transfer. The outcomes of the study were the following: endometrial thickness on the day of progesterone administration, implantation rate, and clinical and viable ongoing pregnancy rates.

**Results:**

Endometrial thickness and clinical outcomes of oral and transdermal estrogen administration were equally successful (*p* > 0.05).

**Conclusion:**

No significant difference was found in endometrial thickness on the day of progesterone administration nor in clinical outcomes between transdermal estrogen and oral estrogen in patients undergoing frozen-thawed single blastocyst stage transfer cycles without GnRHa suppression.

## Introduction

Advances in cryopreservation techniques have resulted in a dramatic increase in freeze-all cycles as the preferred method of ART. One reason for this preference is the higher rate of viable embryos available after thawing, which almost eliminates the risk of ovarian hyperstimulation syndrome (OHSS). Furthermore, many studies have shown increased implantation rates when transfer takes place in a more natural uterine environment without elevated levels of hormones [1–4].

Appropriate endometrial preparation plays an essential role in successful implantation. The two main alternative approaches are estrogen and progesterone replacement treatment or the use of natural cycle, depending on the regularity or pattern of the menstrual cycle and practicalities regarding patient monitoring. Our center’s strategy for endometrial preparation is the use of natural cycle whenever possible. Therefore, our study was undertaken to compare the efficiency of oral and transdermal estrogen replacement treatment in cases where natural cycle is not possible due to anovulation.

There have been several studies comparing the efficiency of different estrogen routes in frozen thawed embryo transfer (FET) cycles. One study compared the effect of estrogen dose and administration route on reproductive outcomes [5]. However, this was conducted retrospectively and in oocyte donation cycles with fresh embryo transfer, and the authors could not exclude the possibility that the response to endometrial state of a high-quality female gamete might be different to that of a patient’s own oocytes.

A meta-analysis by Glujovsky et al. reviewed studies investigating endometrial preparation for women undergoing embryo transfer with frozen embryos or embryos derived from donor oocytes [6]. It concluded that the type of estrogen supplementation and route of administration had no effect on the success rates of FET cycles. However, in these studies gonadotrophin-releasing hormone agonists (GnRHas) were frequently used to prevent the possibility of spontaneous ovulation, whereas in our study, no GnRHas were used. Furthermore, there were no donor cycles.

Thus, our rationale for conducting this study was that, unlike any other study, we were able to find in the literature; it combined all the following factors; no GnRHa pre-treatment was given before estrogen usage; only non-donor cycles were included; embryos were vitrified at blastocyst stage only; in all cases, a single embryo was transferred.

## Materials and methods

### Study population and design

This study was conducted between May 2017 and October 2017 (ClinicalTrials.gov identifier: NCT03155048) in the Assisted Reproductive Technologies and Reproductive Genetics Centre at Istanbul Memorial Hospital with the approval of the Local Ethics Committee. The RCT was designed as a non-inferiority study, as the aim was to investigate whether the estrogen patch was as efficient as oral estrogen for preparation of FET cycles.

Patients having at least one day 5 (*n* = 290) or day 6 (*n* = 12) vitrified blastocyst were informed and written informed consent was signed. The primary outcome measure in these patients with irregular menses or anovulatory cycles undergoing frozen thawed single blastocyst transfer cycles without GnRHa suppression was endometrial thickness on the day of progesterone administration. The secondary outcome measures were implantation rate and clinical outcomes. Exclusion criteria were the following: female age above 38 years, two or more previous unsuccessful cycles, a history of two or more early pregnancy losses, severe endometriosis, severe uterine malformation, azoospermia, and a history of familial thrombophilia or an abnormality in the thrombophilia tests. In addition, patients with polycystic ovarian syndrome with more than 30 cumulus oocyte complexes retrieved at the pick-up were considered to be ineligible and therefore were excluded. The reason for this exclusion is that, this high number of oocytes may be associated with poor oocyte quality and lower rates of blastocyst development and may affect clinical outcomes.

A good prognosis group was targeted for the study. The main reason for the study being restricted to good prognosis patients was that embryos suitable for freezing at the blastocyst stage are normally available from good prognosis patients rather than from poor prognosis patients and this was the case in our study. Furthermore, including only good prognosis patients excluded any possible bias in the study which could have resulted from compromised clinical characteristics of patients.

OHSS-free clinic strategy is applied in our clinic. Accordingly, in patients with irregular menses and anovulation, the main reason for freezing blastocysts was the possible risk of OHSS. Additional reasons for freezing were endometrial polyps, the presence of hydrosalpinx, and patient desire for embryo pooling.

### Estrogen replacement cycles

In 146 patients, 3.9-mg estradiol transdermal patch (Climara®, Bayer Turk, Turkey) was used, whereas in 156 cases, endometrial preparation was done with a fixed dose of 2 mg three times per day oral of estradiol tablets (total 6 mg) (Estrofem®, Novo Nordisk, Denmark).

Estrogen patch or oral administration was started from the second day of menstruation after a basal ultrasonography to rule out the presence of ovarian cysts or other pelvic pathologies. Hormonal evaluation was not carried out if any ovarian cyst(s) were present or if endometrial thickness was more than 5.9 mm. USG scan was repeated on day 10 of the cycle to monitor endometrial thickness and to observe whether there was a spontaneously growing dominant follicle. If there was a dominant follicle, the cycle was to be allowed to continue as a natural cycle and considered to be a drop-out. However, no such event occurred. Otherwise, estrogen administration was continued to day 15 and a further USG scan was performed. If endometrial thickness was 7 mm or more, progesterone vaginal gel (Crinone® 8%; Merck Serono, Switzerland) twice a day, was started on the same day. Otherwise, estrogen administration was continued to day 21. In cases where development had continued satisfactorily, embryo transfer was carried out 5 days after the commencement of progesterone administration. On the morning of the day of transfer, vitrified blastocysts were thawed and cultured in the culture medium (Life Global®, Brussels, Belgium), supplemented with 10% plasmanate (Life Global®, Brussels, Belgium) for 3 to 4 h to observe re-expansion and viability.

### Embryo vitrification and thawing

Good or top-quality blastocysts (at least 3BB) were vitrified on day 5 and day 6 morning with Kitazato vitrification media according to manufacturer’s instructions, using cryotops® as carriers. Blastocysts were thawed with Kitazato warming media according to manufacturer’s instructions. Embryos were first checked for survival 30 min after thawing. A second check occurred 2 h after warming for re-expansion, hatching, extensive cytoplasmic granulation, and the presence of necrotic foci, which are predictors of the rates of implantation, pregnancy, and live birth [7]. Eligible blastocysts with at least 80% re-expansion and vitality were transferred in the afternoon of the same day.

### Embryo scoring

Blastocysts were scored before vitrification according to Gardner’s classification (114–120 h post-ICSI) and classified into three groups: top quality (TQ), good quality (GQ), and poor quality (PQ) blastocysts. The TQ designation includes 3AA, 4AA, and 5AA blastocysts, whereas GQ comprises those graded as 3/4/5BB, AB or BA. Blastocysts of inferior quality were designated as PQ blastocysts.

After embryo transfer, while estrogen administration was continuing either by oral or transdermal route, for luteal phase support, patients received a twice daily dose of progesterone gel administered intravaginally (Crinone® 8%; Merck Serono, Switzerland). Nine days after blastocyst transfer, serum β-hCG was measured. When pregnancy occurred, the same daily doses of estrogen were continued until the 10th week of gestation and of progesterone until the 12th week of gestation. At 7 weeks, a transvaginal ultrasound was performed to monitor early pregnancy. A viable pregnancy was defined as the presence of fetal heart beat and ongoing pregnancy was defined as a 12-week viable pregnancy.

### Randomization

A total number of 317 women undergoing FET cycles were assessed for eligibility and enrolled in the trial once they applied for a thawing cycle. They were randomly assigned to two groups in a ratio of 1:1 by means of computer-generated random numbers (http://www.randomization.com).

### Power analysis

A pilot study, which included 60 patients with 30 in each arm, was designed and entered on ClinicalTrials.gov. A power analysis was conducted according to the results obtained from the pilot study in order to determine the necessary sample size, using the G*Power (v3.1.9) program. Based on the values of endometrial thickness, mean thickness value was found to be 9.39 and 10.04 for the estradiol patch group and estradiol valerate group respectively. According to the evaluation performed using these data, the effect size was calculated to be *W* = 0.397, and each group should include 101 people and a total of 202 people to obtain 80% power at a level of *α* = 0.05. Considering that data loss could occur during the study, it was decided to perform the study with at least 220 patients.

### Statistical analysis

NCSS (Number Cruncher Statistical System) 2007 (Kaysville, Utah, USA) was used for statistical analysis. Regarding the comparisons of quantitative data as well as descriptive statistical methods (mean, standard deviation, median frequency, ratio, minimum, and maximum), Student’s *t* test was used for the intergroup comparisons of quantitative data with normal distribution, and Mann-Whitney *U* test was used for the intergroup comparisons of variables without normal distributions. Pearson’s chi-square test was used for the comparisons of the qualitative data. A *p* value of *p* < 0.05 was accepted as statistically significant.

### Participant flowchart

**Fig. 1:**
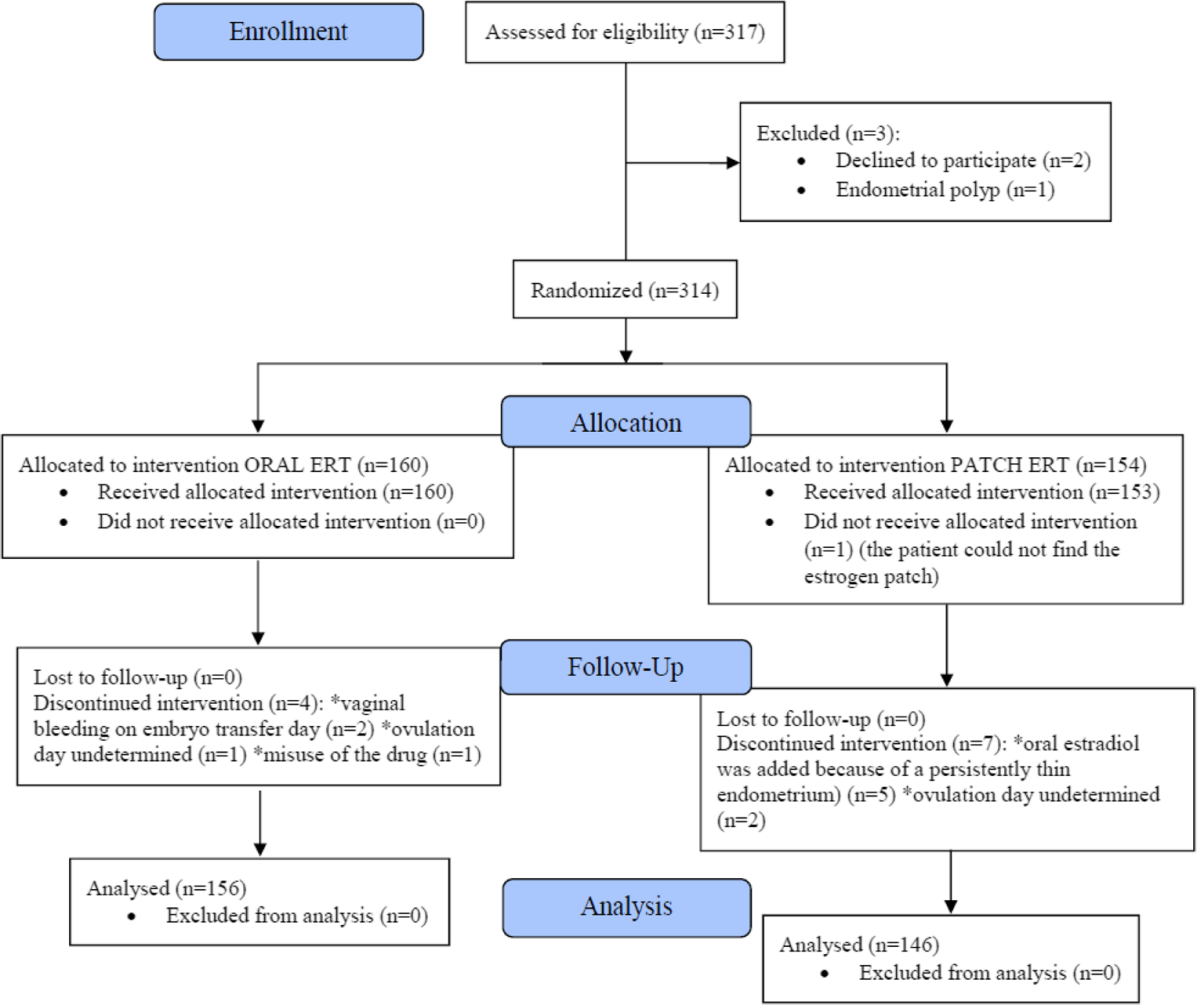
CONSORT flowchart of the trial

A total of 317 patients undergoing FET cycles for frozen-thawed blastocyst transfer were assessed for eligibility. Three patients were excluded before randomization: one patient with endometrial polyps was excluded for hysteroscopy and two other patients declined to participate. Three hundred fourteen patients were randomized: 160 were allocated to oral estrogen replacement therapy (ERT) intervention and 154 patients were allocated to patch ERT intervention. Of the latter group, one patient did not receive intervention because she was unable to obtain the estrogen patch (Fig. 1).

During the course of the study, a total of seven patients were diagnosed as having a thin endometrium, despite no history of this condition. In the oral estrogen group, two women 2/160 (1.25%) did not have embryo transfer because of vaginal bleeding on embryo transfer day, indicating insufficient endometrial thickness. In the transdermal patch group, oral estradiol was added to the treatment of 5/154 (3.24%) women because of a persistently thin endometrium as indicated by transvaginal ultrasonography. However, in both groups, this difference did not reach statistical significance (*p* = 0.230). In addition, the number of participants in this study (*n* = 317) was far in excess of the number indicated as sufficient in the power analysis (*n* = 220). Since inclusion would make no statistically significant difference to the results, it was decided to exclude these seven patients from the evaluation **(**Table 1**)**.

**Table 1:** Demographics of patients undergoing oral or patch ERT cycles

		Total (*n* = 302)	Oral ERT (*n* = 156)	Patch ERT (*n* = 146)	*p*
Infertility diagnoses (% distribution)	Female factor	92 (30.5)	48 (30.8)	44 (30.1)	^a^0.348
	Male factor	97 (32.1)	51 (32.7)	46 (31.5)	
	Unexplained	60 (19.9)	35 (22.4)	25 (17.1)	
	Combined	53 (17.5)	22 (14.1)	31 (21.2)	
Female age	Min-max (median)	21–38 (31)	20–38 (31)	21–38 (32)	^b^0.126
	Mean ± se	31.27 ± 3.97	30.77 ± 3.97	31.57 ± 3.93	
BMI (kg/m^2^)	Min-max (median)	16.7–30 (23.7)	17.7–30 (23.8)	16.7–30 (23.6)	^b^0.891
	Mean ± se	23.94 ± 3.35	23.95 ± 3.43	23.91 ± 3.25	
AMH (ng/mL)	Min-max (median**)**	0–23.5 (3.5)	0–23.5 (3.3)	0.1–20.6 (3.8)	^c^0.977
	Mean ± se	4.36 ± 3.86	4.50 ± 4.25	4.21 ± 3.38	
Endometrial thickness (mm)	Min-max (median)	7–16 (9.6)	7.3–16 (9.7)	7–14 (9.7)	^b^0.651
	Mean ± se	10.03 ± 1.58	10.12 ± 1.72	9.93 ± 1.40	
Patients with endometrial thickness ˂ 7 mm on the 15th day of artificial endometrial preparation cycle (*n*)	*n*	7	2/160 (1.25%)	5/154 (3.24%)	^a^0.230

^a^Pearson chi-squaret, ^b^Student’s *t* test, ^c^Mann-Whitney *U* test

*se* standard error

A total of 302 patients was analyzed, 156 in the oral arm and 146 in the patch arm. The demographic variables were comparable between the oral and the patch estrogen groups (*p* > 0.05). The causes of infertility were female factor only in 30.5% (*n* = 92) of patients, male factor only in 32.1% (*n* = 97) of patients, and combined female and male factor in 17.5% (*n* = 53) and unexplained infertility in 19.9% (*n* = 60) of patients. There was no statistically significant difference in the causes of infertility between the two arms of the study (*p* = 0.348). The patients were between 21 and 38 years of age, the mean age was 31.27 ± 3.97 years. Again, no statistical difference existed between the groups (*p* = 0.126). The mean BMI was 23.94 ± 3.35 kg/m^2^; the mean AMH level was 4.36 ± 3.86 ng/mL, and the mean endometrial thickness (*p* = 0.651) on the day of progesterone administration was 10.03 ± 1.58 mm. None of those values were statistically different between the two groups (Table 1).

The study population consisted of good prognosis patients with a mean of 16.84 retrieved oocytes, 14.25 metaphase II oocytes, 11.83 fertilized oocytes, and 6.22 vitrified blastocysts. A single thawed blastocyst was transferred to each of the analyzed 302 patients. Ninety-six percent of the patients (*n* = 290) had a day 5 blastocyst and 4% (*n* = 12) had a day 6 blastocyst. Blastocyst quality was not statistically significant in either group: 67.9% of the blastocysts were of top quality and 25.6% of good quality in the oral ERT group, and 63.7% of the blastocysts were of top quality and 30.8% of good quality in the patch ERT group (*p* = 0.596) (Table 2).

The biochemical pregnancy rate was higher in the oral ERT group, when compared to the patch group (75.6% vs. 67.1%), although it did not reach statistical significance (*p* = 0.101). The biochemical pregnancy loss was higher in the patch group (9.2% vs. 7.6% in the oral ERT group), but without reaching statistical significance (*p* = 0.680). The viable clinical pregnancy rate was also higher in the oral ERT group (69.9% vs.61%), although it did not reach statistical significance (*p* = 0.103). The clinical miscarriage rate was higher in the oral ERT group (19.3% vs. 14.6% in the patch group) (*p* = 0.387). Finally, the viable ongoing pregnancy rate was 56.4% in the oral ERT group and 52.1% in the patch group (*p* = 0.448). The overall twinning rate after eSET was 0.57% (Table 2).

## Discussion

### Design

Although in our center, natural cycle is the recommended option in FET cycles, it is not always appropriate for all individuals, such as patients with irregular menses or anovulatory cycles. In these cases, up until May 2017, we administered estrogen replacement treatment orally. As far as we know, there is no report in the literature of a randomized clinical trial comparing oral and transdermal estrogen administration in terms of endometrial thickness on the day of progesterone administration and clinical outcomes in patients undergoing frozen-thawed single blastocyst stage transfer cycles without GnRHa suppression in non-donor cycles.

The RCT was designed as a non-inferiority study, as the aim was to investigate whether the estrogen patch, which is indicated in the literature to be safer and is generally regarded as more patient-friendly, less stressful, and less costly, is as efficient as oral estrogen for preparation of FET cycles [8].

The reason for the study being restricted to good prognosis patients were embryos suitable for freezing at the blastocyst stage are normally available from good prognosis patients rather than from poor prognosis patients and this was the case in our study; furthermore, including only good prognosis patients excluded any possible bias in the study which could have resulted from compromised clinical characteristics of patients.

Regarding exclusion criteria, patients with polycystic ovarian syndrome with more than 30 cumulus oocyte complexes retrieved at the pick-up were considered to be ineligible and therefore were excluded. The reason for this exclusion is that this high number of oocytes may be associated with poor oocyte quality and lower rates of blastocyst development and may affect clinical outcomes. Some studies in the literature indicate that folliculogenesis in the PCOS is often disrupted, leading to suboptimal oocyte competence for fertilization [9]. Also, according to a study conducted by Dumesic et al, PCOS-related endocrine/paracrine abnormalities alter the intrafollicular environment and affect granulosa cell-oocyte interactions crucial for achievement of developmental competence of the oocyte [10]. Furthermore, any abnormality in the extra-ovarian and/or intra-ovarian factors may negatively affect the granulosa cell-oocyte interaction, oocyte maturation, and potential embryonic developmental competence, contributing to unsuccessful outcomes for patients with PCOS who are undergoing assisted reproduction [11]. We therefore designed a randomized clinical trial of two different routes, oral or patch, in order to compare their effectiveness in terms of endometrial thickness.

### Findings of previous studies

Although there are studies comparing the effect of true natural and modified natural cycle on clinical outcomes, as far as we know, there is only one randomized controlled study in the literature comparing the effects of oral and transdermal estrogen [12]. Furthermore, the number of patients included in that study was limited, with 45 patients in each arm; embryos were vitrified on the second or third days after retrieval and one to three (mean 2.4) embryos were transferred. In our prospective randomized clinical trial, a total of 317 patients were included, of which 302 were analyzed, the largest study comparing the efficiency of transdermal and oral estrogen in non-donor cycles. Moreover, embryos were vitrified at blastocyst stage and single embryo transfer was the strategy.

An updated Cochrane Database of Systematic Review, Ghobara et al. included randomized controlled trials comparing various cycle regimens and different methods used to prepare the endometrium during FET [13]. Eighteen RCTs were evaluated for FET cycles in 3815 women, and natural cycles, artificial cycle regimens with estrogen, and progesterone or ovulation induction cycles with gonadotropins were compared. Different endometrial preparation methods, true or modified natural cycle, or other FET regimens for endometrial preparations with clomiphene and gonadotropins versus hormonal treatment (HT) FET cycles with or without GnRHa pretreatment were compared. The conclusion was that HT alone was associated with a lower live birth rate than HT with GnRHa suppression. This review concluded that no evidence had been found to support the use of one cycle regimen in preference to another in preparation for frozen thawed embryo transfer in women with regular ovulatory cycles.

Powers et al. showed that levels of circulating estradiol are relevant to efficacy, and that excessively high levels of estrone after oral administration of estrogens merely represent a non-physiologic precursor or metabolite [14].

Burks and Paulson reported that oral estrogen is metabolized both in the intestines and liver and converted to estrone and estrone sulfate with steady-state estrone levels two to six times higher than those of estradiol, whereas the transdermal route circumvents the hepatic metabolism and produces the most stable steady-state levels of estradiol [15]. Therefore, significantly less of the transdermally absorbed E_2_ is converted to the weaker E_1._ Hence, authors hypothesized that the endometrium may react in a different way to an altered mixture of estrogenic compounds including the estrogenic milieu.

Tehraninejad et al. published an RCT to investigate the effects of transdermal estrogen on pregnancy rates of candidates for FET cycle [8]. Oral and transdermal estrogen groups were compared after suppression with GnRHa. Although the clinical pregnancy rates were not significantly different, the miscarriage rate was significantly lower, and ongoing pregnancy and live birth rates were significantly higher in the transdermal estrogen group. Authors concluded that transdermal estrogen showed better results than oral estrogen and can be used safely in patients with a high risk of deep vein thrombosis, hyperlipidemia, and clotting disturbances.

However, for the purposes of our study into endometrial thickness and cycle outcomes of two different routes, only cases without thrombophilia risk factors were included. The following factors were therefore evaluated: smoking habits, diabetes mellitus, kidney disease, obesity, and family history of thrombophilia. The genetic and biochemical panels for thrombophilia were also evaluated.

Krasnow et al. compared the effects of oral micronized estradiol with transdermal estradiol on endometrial receptivity in women undergoing oocyte donation to evaluate endometrial histology and beta-3-integrin expression and concluded that transdermal estradiol therapy results in more physiological circulating estradiol levels and improved endometrial histology compared with daily oral micronized estradiol [16].

During the course of the study, a total of seven patients were diagnosed as having a thin endometrium, despite no history of this condition. In the oral estrogen group, two women 2/160 (1.25%) did not have embryo transfer because of vaginal bleeding on embryo transfer day, indicating insufficient endometrial thickness. In the transdermal patch group, oral estradiol was added to the treatment of 5/154 (3.24%) women because of a persistently thin endometrium as indicated by transvaginal ultrasonography. This could have been because transdermal estrogen application can cause fluctuations in estrogen concentrations, and it may sometimes be difficult to maintain a constant steroid level. However, in both groups, this difference did not reach statistical significance (*p* = 0.230). In addition, the number of participants in this study (*n* = 317) was far in excess of the number indicated as sufficient in the power analysis (*n* = 220). Since inclusion would make no statistically significant difference to the results, it was decided to exclude these seven patients from the evaluation (Table 2).

**Table 2:** Clinical outcomes of ERT cycles

		Total (*n* = 302)	Oral ERT (*n* = 156)	Patch ERT (*n* = 146)	*p*
Outcomes of fresh cycles	Retrieved oocytes @ OPU (mean ± SD)	16.84 ± 9.17	17.58 ± 9.97	16.02 ± 8.15	^a^0.13
	Metaphase II oocytes (mean ± SD)	14.25 ± 7.99	14.94 ± 8.52	13.49 ± 7.30	^a^0.14
	ICSI-fertilized oocytes (mean ± SD)	11.83 ± 6.86	12.27 ± 7.25	11.33 ± 6.38	^a^0.25
	Vitrified blastocyts (mean ± SD)	6.22 ± 4.17	6.25 ± 4.26	6.19 ± 4.08	^a^0.90
Embryo transfer day	Day 5 (*n*, %)	290 (96.0)	151 (96.8)	139 (95.2)	^b^0.480
	Day 6 (*n*, %)	12 (4.0)	5 (3.2)	7 (4.8)	
Blastocyst quality	Poor quality blastocysts (*n*, %)	18 (6.0)	10 (6.4)	8 (5.5)	^b^0.596
	Good quality blastocysts (*n*, %)	85 (28.1)	40 (25.6)	45 (30.8)	
	Top quality blastocysts (*n*, %)	199 (65.9)	106 (67.9)	93 (63.7)	
IVF outcomes	Biochemical pregnancy (*n*, %)	216 (71.5)	118 (75.6)	98 (67.1)	^b^0.101
	Biochemical pregnancy loss (*n*, %)	18 (8.3)	9 (7.6)	9 (9.2)	^b^0.680
	Viable clinical pregnancy (*n*, %)	198 (65.6)	109 (69.9)	89 (61.0)	^b^0.103
	Clinical miscarriage (*n*, %)	34 (17.2)	21 (19.3)	13 (14.6)	^b^0.387
	Viable ongoing pregnancy (*n*, %)	164 (54.3)	88 (56.4)	76 (52.1)	^b^0.448

^a^Student’s *t* test, ^b^Pearson chi-square test

Mackens et al. stated that the rate of early pregnancy loss is alarmingly high in some reports probably as a result of an embryo-endometrium asynchrony and that, therefore, caution when using HRT for FET is necessary [17, 18]. In our study, however, neither biochemical nor clinical pregnancy losses were found to be higher than in fresh transfers. However, compared to natural cycles, which is the main strategy in our center for the ovulatory cases, a significantly higher number of early pregnancy losses were observed with HR treatment (unpublished data).

In conclusion, no significant difference was found in endometrial thickness on the day of progesterone administration nor in clinical outcomes between transdermal estrogen and oral estrogen in patients undergoing frozen-thawed single blastocyst stage transfer cycles without GnRHa suppression.

## References

[1] Wong KM, Mastenbroek S, Repping S (2014). Cryopreservation of human embryos and its contribution to in vitro fertilization success rates. Fertil Steril.

[2] Roque M, Lattes K, Serra S, Solà I, Geber S, Carreras R, Checa MA (2013). Fresh embryo transfer versus frozen embryo transfer in in vitro fertilization cycles: a systematic review and meta-analysis. Fertil Steril.

[3] Roque M, Valle M, Guimarães F, Sampaio M, Geber S (2015). Freeze-all policy: fresh vs. frozen-thawed embryo transfer. Fertil Steril.

[4] Zhu Q, Chen Q, Wang L, Lu X, Lyu Q, Wang Y, Kuang Y (2018). Live birth rates in the first complete IVF cycle among 20 687 women using a freeze-all strategy. Hum Reprod.

[5] Madero S, Rodriguez A, Vassena R, Vernaeve V (2016). Endometrial preparation: effect of estrogen dose and administration route on reproductive outcomes in oocyte donation cycles with fresh embryo transfer. Hum Reprod.

[6] Glujovsky D, Pesce R, Fiszbajn G, Sueldo C, Hart RJ, Ciapponi A. Endometrial preparation for women undergoing embryo transfer with frozen embryos or embryos derived from donor oocytes. Cochrane Database Syst Rev. 2010;(1):CD006359. 10.1002/14651858.CD006359.pub2 20091592

[7] Ebner T, Vanderzwalmen P, Shebl O, Urdl W, Moser M, Zech NH, Tews G (2009). Morphology of vitrified/warmed day-5 embryos predicts rates of implantation, pregnancy and live birth. Reprod BioMed Online.

[8] Shahrokh Tehraninejad E, Kabodmehri R, Hosein Rashidi B, Jafarabadi M, Keikha F, Masomi M, Hagholahi F (2018). Transdermal estrogen (oestrogel) for endometrial preparation in freeze embryo transfer cycle: An RCT. Int J Reprod Biomed (Yazd).

[9] Patel SS, Carr BR (2008). Oocyte quality in adult polycystic ovary syndrome. Semin Reprod Med.

[10] Dumesic DA, Padmanabhan V, Abbott DH (2008). Polycystic ovary syndrome and oocyte developmental competence. Obstet Gynecol Surv.

[11] Qiao J, Feng HL (2011). Extra- and intra-ovarian factors in polycystic ovary syndrome: impact on oocyte maturation and embryo developmental competence. Hum Reprod Update.

[12] Davar R, Janati S, Mohseni F, Khabazkhoob M, Asgari SA (2016). Comparison of the effects of transdermal estradiol and estradiol valerate on endometrial receptivity in frozen-thawed embryo transfer cycles: a randomized clinical trial. J Reprod Infertil.

[13] Ghobara T, Gelbaya TA, Ayeleke RO (2017). Cycle regimens for frozen-thawed embryo transfer. Cochrane Database Syst Rev.

[14] Powers MS, Schenkel L, Darley PE, Good WR, Balestra JC, Place VA (1985). Pharmacokinetics and pharmacodynamics of transdermal dosage forms of 17β-estradiol: comparison with conventional oral estrogens used for hormone replacement. Am J Obstet Gynecol.

[15] Burks H, Paulson R (2015). Cryopreserved embryo transfer: endometrial preparation and timing. Semin Reprod Med.

[16] Krasnow JS, Lessey BA, Naus G, Hall LL, Guzick DS, Berga SL (1996). Comparison of transdermal versus oral estradiol on endometrial receptivity. Fertil Steril.

[17] Mackens S, Santos-Ribeiro S, van de Vijver A, Racca A, Van Landuyt L, Tournaye H, Blockeel C (2017). Frozen embryo transfer: a review on the optimal endometrial preparation and timing. Hum Reprod.

[18] Wilcox AJ, Baird DD, Weinberg CR (1999). Time of implantation of the conceptus and loss of pregnancy. N Engl J Med.

